# Supplementary figures and images for: Patterns of respiratory health services utilization from birth to 5 years of children who experienced adverse birth outcomes
Source: PLoS One. 2021 Feb 19;16(2):e0247527. doi: 10.1371/journal.pone.0247527 (PMC7895380; doi:10.1371/journal.pone.0247527)

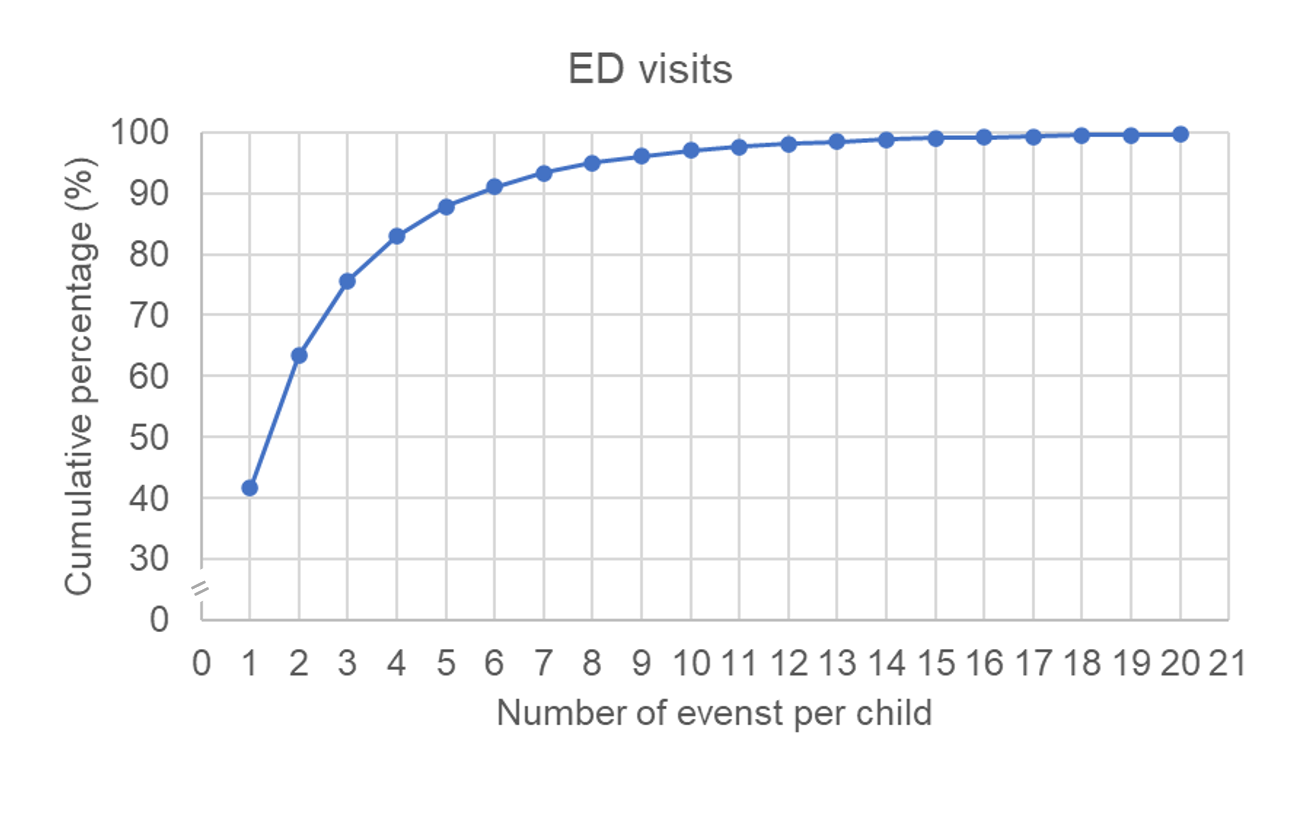

Supplement: S1 Fig — (TIFF) [file pone.0247527.s002.tiff]

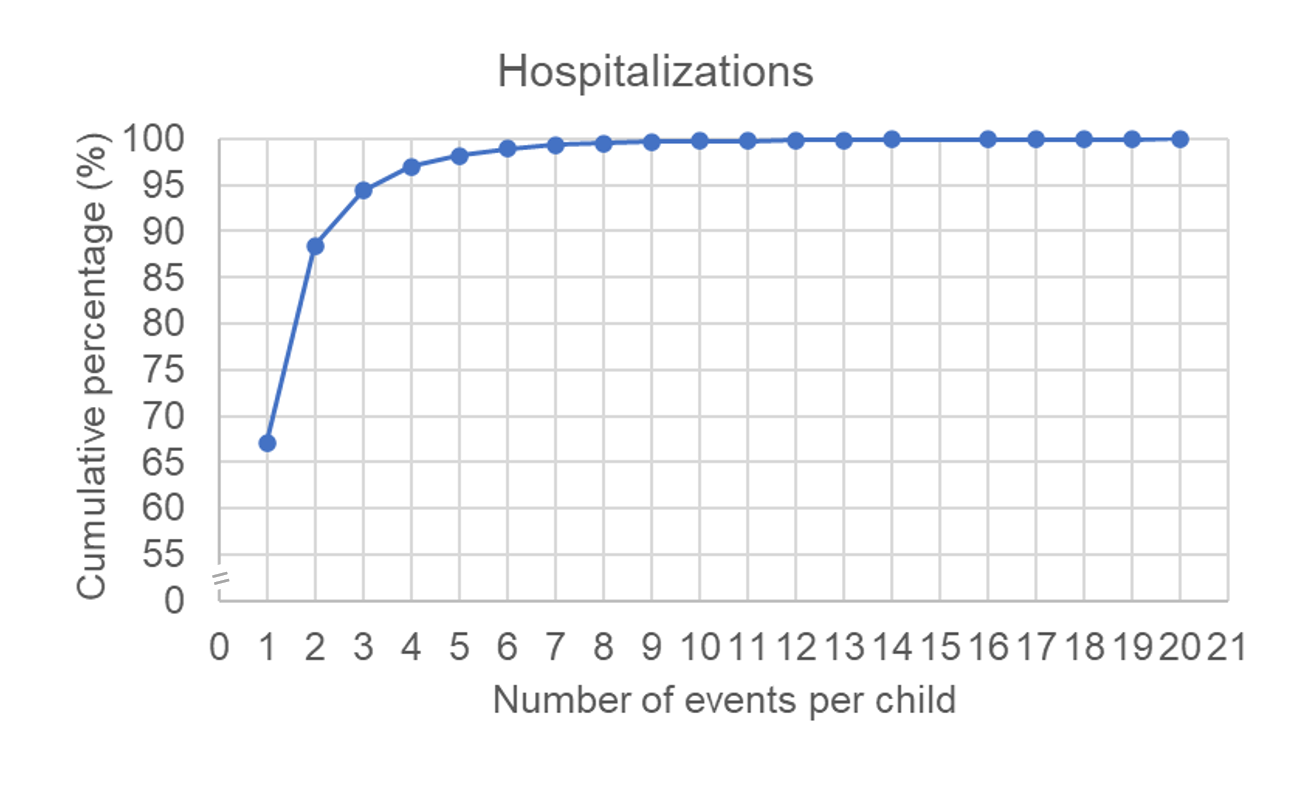

Supplement: S2 Fig — (TIFF) [file pone.0247527.s003.tiff]
