# Supplementary material for: Patterns of respiratory health services utilization from birth to 5 years of children who experienced adverse birth outcomes
Source: PLoS One. 2021 Feb 19;16(2):e0247527. doi: 10.1371/journal.pone.0247527 (PMC7895380; doi:10.1371/journal.pone.0247527)
Supplement: S1 Table — (PDF) [file pone.0247527.s004.pdf]

S1 Table. Rate ratios for respiratory hospitalizations and ED visits from birth to five years for adverse birth groups.

Adverse birth group	Outcome	Hospitalizations RR [95% CI]		Ed visits RR [95% CI]	
		Unadjusted	Adjusted	Unadjusted	Adjusted
Moderate/late PTB	Asthma	1.78 [1.55, 2.05]	**1.78** [1.53, 2.08]	1.48 [1.38, 1.60]	**1.53** [1.42, 1.66]
	Bronchiolitis	2.18 [1.98, 2.40]	**2.17** [1.96, 2.40]	1.71 [1.60, 1.82]	**1.67** [1.56, 1.80]
	Bronchitis	1.79 [1.25, 2.57]	**1.88** [1.28, 2.78]	1.18 [1.09, 1.26]	**1.20** [1.11, 1.30]
	Croup	1.41 [1.13, 1.76]	**1.52** [1.20, 1.92]	1.21 [1.14, 1.29]	**1.25** [1.17, 1.33]
	Influenza	1.87 [1.37, 2.55]	**1.81** [1.29, 2.54]	1.26 [1.12, 1.41]	**1.31** [1.15, 1.49]
	Other LRTIs	2.33 [1.73, 3.14]	**2.41** [1.77, 3.28]	1.36 [1.20, 1.54]	**1.35** [1.19, 1.55]
	Other URTIs	1.83 [1.57, 2.14]	**1.88** [1.60, 2.22]	1.12 [1.08, 1.16]	**1.15** [1.10, 1.19]
	Pneumonia	1.70 [1.52, 1.90]	**1.69** [1.50, 1.89]	1.43 [1.35, 1.52]	**1.50** [1.41, 1.60]
Very PTB	Asthma	4.29 [3.25, 5.68]	**3.98** [2.73, 5.81]	2.26 [1.96, 2.60]	**2.43** [2.01, 2.93]
	Bronchiolitis	4.25 [3.51, 5.14]	**3.59** [2.89, 4.47]	2.17 [1.89, 2.49]	**2.20** [1.86, 2.61]
	Bronchitis	2.45 [1.22, 4.90]	**3.83** [1.62, 9.09]	1.38 [1.14, 1.66]	**1.35** [1.05, 1.73]
	Croup	3.48 [2.08, 5.83]	**3.26** [1.94, 5.48]	1.23 [1.06, 1.42]	**1.24** [1.04, 1.48]
	Influenza	6.12 [4.22, 8.89]	**2.33** [1.30, 4.18]	0.94 [0.75, 1.18]	0.89 [0.67, 1.20]
	Other LRTIs	8.91 [6.33, 12.54]	**4.81** [2.90, 7.97]	1.26 [0.94, 1.70]	1.08 [0.77, 1.52]
	Other URTIs	3.42 [2.73, 4.28]	**2.69** [1.91, 3.78]	0.97 [0.88, 1.06]	0.98 [0.88, 1.10]
	Pneumonia	4.17 [3.31, 5.24]	**3.33** [2.54, 4.38]	2.42 [2.13, 2.75]	**2.26** [1.90, 2.69]
SGA at term	Asthma	1.09 [0.93, 1.31]	1.10 [0.92, 1.31]	1.06 [0.97, 1.16]	1.05 [0.96, 1.15]
	Bronchiolitis	1.14 [1.02,1.28]	**1.13** [1.01, 1.27]	1.05 [0.97, 1.13]	1.04 [0.97, 1.12]
	Bronchitis	1.34 [0.87, 2.09]	1.33 [0.86, 2.07]	0.97 [0.89, 1.06]	0.98 [0.89, 1.06]
	Croup	0.85 [0.63, 1.14]	0.85 [0.63, 1.15]	0.87 [0.82, 0.92]	**0.86** [0.82, 0.92]
	Influenza	1.35 [0.97, 1.88]	1.33 [0.95, 1.86]	1.00 [0.92, 1.10]	1.00 [0.91, 1.09]
	Other LRTIs	1.27 [0.86, 1.89]	1.24 [0.83, 1,83]	1.16 [1.02, 1.33]	**1.16** [1.01, 1.32]
	Other URTIs	1.26 [1.06, 1.50]	**1.25** [1.05, 1.49]	1.03 [0.99, 1.06]	1.02 [0.99, 1.06]
	Pneumonia	1.09 [0.95, 1.26]	1.09 [0.94, 1.25]	1.03 [0.95, 1.10]	1.02 [0.95, 1.10]
LGA at term	Asthma	0.91 [0.78, 1.05]	0.92 [0.78, 1.08]	1.00 [0.92, 1.09]	1.00 [0.93, 1.09]
	Bronchiolitis	1.14 [1.03, 1.27]	**1.14** [1.02, 1.26]	1.03 [0.96, 1.10]	1.03 [0.96, 1.11]
	Bronchitis	1.86 [1.34, 2.57]	**1.86** [1.35, 2.57]	0.97 [0.90, 1.04]	0.98 [0.91, 1.05]
	Croup	1.24 [0.98, 1.57]	1.24 [0.99, 1.57]	1.17 [1.11, 1.24]	**1.17** [1.11, 1.24]
	Influenza	1.22 [0.85, 1.73]	1.22 [0.85, 1.74]	0.98 [0.89, 1.08]	0.98 [0.89, 1.09]
	Other LTRIs	1.16 [0.83, 1.62]	1.15 [0.82, 1.61]	1.01 [0.91, 1.12]	1.01 [0.91, 1.11]
	Other URTIs	0.86 [0.73, 1.02]	0.86 [0.72, 1.02]	1.03 [1.01, 1.04]	**1.03** [1.01, 1.04]
	Pneumonia	0.96 [0.85, 1.08]	0.95 [0.84, 1.07]	0.99 [0.93, 1.05]	0.99 [0.93, 1.05]

CI = confidence interval; LGA = large for gestational age; LRIs = lower respiratory tract infections; URTIs = upper respiratory tract infections; PTB= preterm birth; RR = rate ratios; SGA = small for gestational age.

RR adjusted by sex, 5, minute Apgar score, bronchopulmonary dysplasia, use of significant resuscitation methods, and material and social deprivation. Reference group = appropriate-for-gestational age infants born at term.
