# Supplementary material for: Patterns of respiratory health services utilization from birth to 5 years of children who experienced adverse birth outcomes
Source: PLoS One. 2021 Feb 19;16(2):e0247527. doi: 10.1371/journal.pone.0247527 (PMC7895380; doi:10.1371/journal.pone.0247527)
Supplement: S1 Appendix — (PDF) [file pone.0247527.s005.pdf]

S1 Appendix. Age-specific trajectories of respiratory health services utilization for single respiratory conditions by birth groups.

Table A. Age-specific trajectories of health service utilization due to asthma by birth groups**.**

Asthma			
**Age (years)**	**Birth group**	**Predicted rate (per 1000 per year)**	**95% CI**
0 to <1	Reference group	14.8	[14.2, 15.4]
	Moderate/late PTB	25.8	[23.0, 28.6]
	Very PTB	40.5	[32.0, 48.9]
	SGA at term	16.5	[14.6, 18.5]
	LGA at term	17.5	[15.6, 19.5]
1 to <2	Reference group	27.6	[26.7, 28.4]
	Moderate/late PTB	49.0	[45.1, 52.9]
	Very PTB	93.8	[80.6, 107.0]
	SGA at term	29.1	[26.5, 31.8]
	LGA at term	28.9	[26.4, 31.4]
2 to <3	Reference group	25.5	[24.7, 26.3]
	Moderate/late PTB	36.7	[33.3, 40.0]
	Very PTB	77.8	[65.9, 89.7]
	SGA at term	26.5	[24.0, 29.0]
	LGA at term	22.9	[20.7, 25.1]
3 to <4	Reference group	21.7	[21.0, 22.5]
	Moderate/late PTB	35.5	[32.2, 38.8]
	Very PTB	48.5	[39.2, 57.8]
	SGA at term	22.6	[20.3, 24.9]
	LGA at term	22.3	[20.1, 24.5]
4 to <5	Reference group	18.4	[17.7, 19.1]
	Moderate/late PTB	28.8	[25.8, 31.7]
	Very PTB	46.2	[37.2, 55.3]
	SGA at term	18.3	[16.2, 20.4]
	LGA at term	17.1	[15.2, 19.0]
5 to <6	Reference group	14.6	[14.0, 15.3]
	Moderate/late PTB	22.8	[20.2, 25.5]
	Very PTB	30.7	[23.3, 38.0]
	SGA at term	16.6	[14.6, 18.6]
	LGA at term	16.6	[14.7, 18.5]

Predicted rates expressed as aggregated ED visits and hospitalizations per 1,000 singleton live births per year, adjusted by sex, 5, minute Apgar score, bronchopulmonary dysplasia, use of significant resuscitation methods, and material and social deprivation.
CI = Confidence intervals; LGA=large for gestational age; PTB=preterm birth; SGA=small for gestational age. Reference group = appropriate-for-gestational age infants born at term.

Table B. Age-specific trajectories of health service utilization due to bronchiolitis by birth groups.

Bronchiolitis			
**Age (years)**	**Birth group**	**Predicted rate (per 1000 per year)**	**95% CI**
0 to <1	Reference group	71.0	[69.6, 72.3
	Moderate/late PTB	138.1	[[131.6, 144.6]
	Very PTB	175.7	[157.1, 194.3]
	SGA at term	73.4	[69.2, 77.5]
	LGA at term	83.8	[79.6, 88.1]
1 to <2	Reference group	24.4	[23.6, 25.2]
	Moderate/late PTB	40.2	[36.7, 43.7]
	Very PTB	76.9	[65.2, 88.6]
	SGA at term	23.9	[21.5, 26.2]
	LGA at term	28.0	[25.5, 30.4]
2 to <3	Reference group	7.5	[7.1, 8.0]
	Moderate/late PTB	11.8	[9.9, 13.7]
	Very PTB	21.5	[15.5, 27.5]
	SGA at term	5.7	[4.5, 6.8]
	LGA at term	7.7	[6.4, 9.0]
3 to <4	Reference group	3.1	[2.8, 3.4]
	Moderate/late PTB	6.3	[4.9, 7.7]
	Very PTB	13.7	[8.9, 18.5]
	SGA at term	3.7	[2.8, 4.7]
	LGA at term	4.5	[3.6, 5.5]
4 to <5	Reference group	2.0	[1.7, 2.2]
	Moderate/late PTB	2.7	[1.8, 3.6]
	Very PTB	3.4	[1.1, 5.8]
	SGA at term	1.8	[1.1, 2.4]
	LGA at term	1.9	[1.2, 2.5]
5 to <6	Reference group	1.3	[1.1, 1.5]
	Moderate/late PTB	1.4	[0.8, 2.1]
	Very PTB	3.0	[0.8, 5.2]
	SGA at term	1.2	[0.7, 1.8]
	LGA at term	0.9	[0.5, 1.3]

Predicted rates expressed as aggregated ED visits and hospitalizations per 1,000 singleton live births per year, adjusted by sex, 5, minute Apgar score, bronchopulmonary dysplasia, use of significant resuscitation methods, and material and social deprivation.
CI = Confidence intervals; LGA=large for gestational age; PTB=preterm birth; SGA=small for gestational age. Reference group = appropriate-for-gestational age infants born at term.

Table C. Age-specific trajectories of health service utilization due to bronchitis by birth groups.

Bronchitis			
**Age (years)**	**Birth group**	**Predicted rate (per 1000 per year)**	**95% CI**
0 to <1	Reference group	17.2	[16.5, 17.8]
	Moderate/late PTB	21.1	[18.6, 23.7]
	Very PTB	18.3	[12.7, 23.9]
	SGA at term	14.2	[12.4, 16.1]
	LGA at term	22.2	[20.0, 24.3]
1 to <2	Reference group	17.8	[17.1, 18.5]
	Moderate/late PTB	21.8	[19.2, 24.4]
	Very PTB	31.8	[24.3, 39.3]
	SGA at term	15.3	[13.4, 17.2]
	LGA at term	19.6	[17.5, 21.6]
2 to <3	Reference group	11.3	[10.8, 11.9]
	Moderate/late PTB	13.3	[11.3, 15.4]
	Very PTB	19.2	[13.4, 24.9]
	SGA at term	9.0	[7.6, 10.4]
	LGA at term	14.0	[12.3, 15.8]
3 to <4	Reference group	9.4	[8.9, 9.9]
	Moderate/late PTB	11.6	[9.7, 13.5]
	Very PTB	12.2	[7.6, 16.8]
	SGA at term	8.1	[6.8, 9.5]
	LGA at term	10.8	[9.3, 12.4]
4 to <5	Reference group	7.4	[7.0, 7.9]
	Moderate/late PTB	9.0	[7.3, 10.6]
	Very PTB	10.9	[6.6, 15.2]
	SGA at term	5.5	[4.4, 6.6]
	LGA at term	8.4	[7.0, 9.7]
5 to <6	Reference group	5.5	[5.1, 5.9]
	Moderate/late PTB	6.9	[5.5, 8.4]
	Very PTB	7.0	[3.5, 10.4]
	SGA at term	4.7	[3.7, 5.8]
	LGA at term	7.1	[5.9, 8.4]

Predicted rates expressed as aggregated ED visits and hospitalizations per 1,000 singleton live births per year, adjusted by sex, 5, minute Apgar score, bronchopulmonary dysplasia, use of significant resuscitation methods, and material and social deprivation.
CI = Confidence intervals; LGA=large for gestational age; PTB=preterm birth; SGA=small for gestational age. Reference group = appropriate-for-gestational age infants born at term.

Table D. Age-specific trajectories of health service utilization due to croup by birth groups.

Croup			
**Age (years)**	**Birth group**	**Predicted rate (per 1000 per year)**	**95% CI**
0 to <1	Reference group	28.3	[27.4, 29.1]
	Moderate/late PTB	35.2	[31.9, 38.5]
	Very PTB	35.6	[27.6, 43.7]
	SGA at term	23.0	[20.7, 25.2]
	LGA at term	31.8	[29.2, 34.4]
1 to <2	Reference group	48.0	[46.9, 49.1]
	Moderate/late PTB	57.7	[53.5, 61.9]
	Very PTB	62.1	[51.4, 72.8]
	SGA at term	42.5	[39.3, 45.7]
	LGA at term	59.4	[55.8, 62.9]
2 to <3	Reference group	34.0	[33.1, 35.0]
	Moderate/late PTB	39.7	[36.2, 43.2]
	Very PTB	44.8	[35.7, 53.8]
	SGA at term	26.7	[24.2, 29.2]
	LGA at term	39.4	[36.5, 42.3]
3 to <4	Reference group	24.0	[23.2, 24.7]
	Moderate/late PTB	30.1	[27.0, 33.1]
	Very PTB	24.2	[17.6, 30.8]
	SGA at term	19.9	[17.7, 22.1]
	LGA at term	30.8	[28.3, 33.4]
4 to <5	Reference group	17.7	[17.0, 18.4]
	Moderate/late PTB	24.3	[21.5, 27.0]
	Very PTB	25.1	[18.4, 31.8]
	SGA at term	13.6	[11.8, 15.4]
	LGA at term	21.6	[19.5, 23.8]
5 to <6	Reference group	11.5	[11.0, 12.1]
	Moderate/late PTB	15.3	[13.2, 17.5]
	Very PTB	16.4	[11.0, 21.9]
	SGA at term	11.6	[10.0, 13.3]
	LGA at term	13.2	[11.5, 14.9]

Predicted rates expressed as aggregated ED visits and hospitalizations per 1,000 singleton live births per year, adjusted by sex, 5, minute Apgar score, bronchopulmonary dysplasia, use of significant resuscitation methods, and material and social deprivation.

CI = Confidence intervals; LGA=large for gestational age; PTB=preterm birth; SGA=small for gestational age. Reference group = appropriate-for-gestational age infants born at term.

Table E. Age-specific trajectories of health service utilization due to influenza by birth groups.

Influenza			
**Age (years)**	**Birth group**	**Predicted rate (per 1000 per year)**	**95% CI**
0 to <1	Reference group	6.7	[6.3, 7.2]
	Moderate/late PTB	13.5	[11.4, 15.5]
	Very PTB	9.0	[5.2, 12.8]
	SGA at term	6.2	[5.0, 7.5]
	LGA at term	8.4	[7.0, 9.7]
1 to <2	Reference group	7.8	[7.3, 8.2]
	Moderate/late PTB	9.4	[7.7, 11.1]
	Very PTB	7.8	[4.3, 11.4]
	SGA at term	7.8	[6.5, 9.2]
	LGA at term	8.7	[7.4, 10.1]
2 to <3	Reference group	6.0	[5.6, 6.4]
	Moderate/late PTB	6.9	[5.4, 8.4]
	Very PTB	7.8	[4.3, 11.4]
	SGA at term	6.3	[5.1, 7.5]
	LGA at term	6.3	[5.1, 7.4]
3 to <4	Reference group	6.0	[5.6, 6.4]
	Moderate/late PTB	7.7	[6.2, 9.2]
	Very PTB	5.9	[2.8, 8.9]
	SGA at term	6.1	[4.9, 7.3]
	LGA at term	5.3	[4.2, 6.3]
4 to <5	Reference group	5.2	[4.8, 5.5]
	Moderate/late PTB	5.9	[4.6, 7.3]
	Very PTB	4.3	[1.7, 6.9]
	SGA at term	4.4	[3.4, 5.4]
	LGA at term	5.0	[3.9, 6.0]
5 to <6	Reference group	3.4	[3.1, 3.7]
	Moderate/late PTB	3.4	[2.4, 4.4]
	Very PTB	2.7	[0.7, 4.8]
	SGA at term	3.1	[2.2, 3.9]
	LGA at term	3.9	[2.9, 4.8]

Predicted rates expressed as aggregated ED visits and hospitalizations per 1,000 singleton live births per year, adjusted by sex, 5, minute Apgar score, bronchopulmonary dysplasia, use of significant resuscitation methods, and material and social deprivation.
CI = Confidence intervals; LGA=large for gestational age; PTB=preterm birth; SGA=small for gestational age. Reference group = appropriate-for-gestational age infants born at term.

Table F. Age-specific trajectories of health service utilization due to other lower respiratory tract infections by birth groups.

Other lower respiratory tract infections			
**Age (years)**	**Birth group**	**Predicted rate (per 1000 per year)**	**95% CI**
0 to <1	Reference group	5.3	[4.9, 5.7]
	Moderate/late PTB	9.0	[7.3, 10.6]
	Very PTB	9.4	[5.7, 13.0]
	SGA at term	5.7	[4.5, 6.8]
	LGA at term	7.4	[6.1, 8.6]
1 to <2	Reference group	5.8	[5.4, 6.2]
	Moderate/late PTB	9.7	[8.0, 11.4]
	Very PTB	8.0	[4.6, 11.4]
	SGA at term	6.3	[5.1, 7.5]
	LGA at term	7.0	[5.8, 8.3]
2 to <3	Reference group	4.1	[3.8, 4.4]
	Moderate/late PTB	5.4	[4.1, 6.7]
	Very PTB	8.0	[4.6, 11.4]
	SGA at term	4.6	[3.5, 5.6]
	LGA at term	4.3	[3.4, 5.3]
3 to <4	Reference group	3.2	[2.9, 3.5]
	Moderate/late PTB	4.0	[2.9, 5.1]
	Very PTB	4.5	[2.0, 7.0]
	SGA at term	3.2	[2.4, 4.1]
	LGA at term	3.5	[2.6, 4.4]
4 to <5	Reference group	2.8	[2.5, 3.1]
	Moderate/late PTB	3.8	[2.7, 4.8]
	Very PTB	4.5	[2.0, 7.0]
	SGA at term	3.3	[2.4, 4.2]
	LGA at term	2.6	[1.8, 3.3]
5 to <6	Reference group	2.0	[1.8, 2.2]
	Moderate/late PTB	3.8	[2.8, 4.9]
	Very PTB	3.1	[1.1, 5.2]
	SGA at term	1.8	[1.2, 2.5]
	LGA at term	2.8	[2.0, 3.5]

Predicted rates expressed as aggregated ED visits and hospitalizations per 1,000 singleton live births per year, adjusted by sex, 5, minute Apgar score, bronchopulmonary dysplasia, use of significant resuscitation methods, and material and social deprivation.
CI = Confidence intervals; LGA=large for gestational age; PTB=preterm birth; SGA=small for gestational age. Reference group = appropriate-for-gestational age infants born at term.

Table G. Age-specific trajectories of health service utilization due to pneumonia by birth groups.

Pneumonia			
**Age (years)**	**Birth group**	**Predicted rate (per 1000 per year)**	**95% CI**
0 to <1	Reference group	30.4	[29.5, 31.3]
	Moderate/late PTB	54.1	[50.0, 58.1]
	Very PTB	86.7	[74.6, 98.8]
	SGA at term	29.9	[27.2, 32.5]
	LGA at term	36.2	[33.5, 39.0]
1 to <2	Reference group	36.7	[35.8, 37.7]
	Moderate/late PTB	55.1	[51.0, 59.2]
	Very PTB	105.2	[91.7, 118.6]
	SGA at term	34.8	[31.9, 37.6]
	LGA at term	41.2	[38.2, 44.2]
2 to <3	Reference group	21.8	[21.1, 22.6]
	Moderate/late PTB	36.2	[32.8, 39.5]
	Very PTB	71.0	[60.2, 81.9]
	SGA at term	20.4	[18.2, 22.6]
	LGA at term	21.8	[19.6, 24.0]
3 to <4	Reference group	15.7	[15.1, 16.4]
	Moderate/late PTB	25.8	[23.0, 28.6]
	Very PTB	34.5	[27.1, 41.9]
	SGA at term	15.3	[13.4, 17.2]
	LGA at term	16.7	[14.8, 18.6]
4 to <5	Reference group	11.3	[10.7, 11.8]
	Moderate/late PTB	18.6	[16.2, 20.9]
	Very PTB	35.3	[27.8, 42.8]
	SGA at term	11.6	[9.9, 13.2]
	LGA at term	10.5	[9.0, 12.0]
5 to <6	Reference group	8.1	[7.6, 8.5]
	Moderate/late PTB	12.8	[10.8, 14.8]
	Very PTB	20.1	[14.4, 25.7]
	SGA at term	9.4	[7.9, 10.9]
	LGA at term	9.2	[7.8, 10.6]

Predicted rates expressed as aggregated ED visits and hospitalizations per 1,000 singleton live births per year, adjusted by sex, 5, minute Apgar score, bronchopulmonary dysplasia, use of significant resuscitation methods, and material and social deprivation.
CI = Confidence intervals; LGA=large for gestational age; PTB=preterm birth; SGA=small for gestational age. Reference group = appropriate-for-gestational age infants born at term.

Table H. Age-specific trajectories of health service utilization due to other upper respiratory tract infections by birth groups.

Other upper respiratory tract infections			
**Age (years)**	**Birth group**	**Predicted rate (per 1000 per year)**	**95% CI**
0 to <1	Reference group	194.5	[192.2, 196.7]
	Moderate/late PTB	236.2	[227.6, 244.7]
	Very PTB	180.8	[162.7, 199.0]
	SGA at term	185.9	[179.3, 192.5]
	LGA at term	223.7	[216.8, 230.7]
1 to <2	Reference group	175.3	[173.2, 177.4]
	Moderate/late PTB	208.2	[200.2, 216.3]
	Very PTB	196.7	[177.8, 215.7]
	SGA at term	164.2	[158.1, 170.4]
	LGA at term	200.1	[193.6, 206.7]
2 to <3	Reference group	116.9	[115.2, 118.7]
	Moderate/late PTB	136.0	[129.5, 142.4]
	Very PTB	125.9	[110.8, 140.9]
	SGA at term	110.9	[105.8, 116.0]
	LGA at term	133.9	[128.6, 139.3]
3 to <4	Reference group	98.1	[96.5, 99.7]
	Moderate/late PTB	113.3	[107.4, 119.2]
	Very PTB	103.6	[90.0, 117.2]
	SGA at term	89.1	[84.5, 93.7]
	LGA at term	111.8	[106.9, 116.7]
4 to <5	Reference group	82.5	[81.0, 84.0]
	Moderate/late PTB	92.8	[87.5, 98.2]
	Very PTB	99.5	[86.2, 112.8]
	SGA at term	78.2	[73.9, 82.5]
	LGA at term	90.8	[86.4, 95.2]
5 to <6	Reference group	68.5	[67.1, 69.8]
	Moderate/late PTB	76.4	[71.6, 81.3]
	Very PTB	74.1	[62.6, 85.5]
	SGA at term	62.2	[58.4, 66.1]
	LGA at term	79.2	[75.1, 83.3]

Predicted rates expressed as aggregated ED visits and hospitalizations per 1,000 singleton live births per year, adjusted by sex, 5, minute Apgar score, bronchopulmonary dysplasia, use of significant resuscitation methods, and material and social deprivation.
CI = Confidence intervals; LGA=large for gestational age; PTB=preterm birth; SGA=small for gestational age. Reference group = appropriate-for-gestational age infants born at term.
